# Cardiometabolic Risk Factors in Patients with Erectile Dysfunction

**DOI:** 10.1155/2014/892091

**Published:** 2014-01-22

**Authors:** Serhat Tanik, Savas Sarikaya, Kürşad Zengin, Sebahattin Albayrak, Yunus Keser Yilmaz, Lutfi Akyol

**Affiliations:** ^1^Department of Urology, Bozok University, School of Medicine, Yozgat, Turkey; ^2^Department of Cardiology, Bozok University, School of Medicine, Yozgat, Turkey; ^3^Department of Cardiovascular Surgery, Bozok University, School of Medicine, Yozgat, Turkey; ^4^Department of Internal Medicine, Bozok University, School of Medicine, Yozgat, Turkey

## Abstract

*Introduction*. There is an increasing interest in the association between erectile dysfunction (ED) and cardiovascular risk factor. Epicardial adipose tissue (EAT) is associated with insulin resistance, increased cardiometabolic risk, and coronary artery disease. Our aim was to investigate relationships between epicardial fat thickness (EFT) as a cardiometabolic risk factor and erectile dysfunction. *Method*. We selected 30 erectile dysfunction patients without comorbidities and 30 healthy individuals. IIEF-5 score was applied to all patients, and IIEF-5 score below 22 was considered as erectile dysfunction. EFT was measured by echocardiography. *Results*. Body mass index (BMI) was higher in ED patients than those without ED (28.19 ± 4.45 kg/m^2^ versus 23.84 ± 2.36 kg/m^2^, *P* = 0.001, resp.). Waist circumstance (WC) was higher in ED patients than those without ED (106.60 ± 5.90 versus 87.86 ± 14.51, *P* = 0.001, resp.). EFT was higher in ED patients compared to non-ED patients (0.49 ± 0.09 cm versus 0.45 ± 0.03 cm, *P* = 0.016, resp.). There was positive correlation among BMI, WC, and EFT. There was negative correlation between EFT and IIEF-5 score (*r* : − 0.632, *P* = 0.001). *Conclusion*. EAT, BMI, and WC as cardiometabolic risk factors were higher in erectile dysfunction patients.

## 1. Introduction

There is an increasing interest in the association between erectile dysfunction (ED) and cardiovascular risk factor. ED share, similar modifiable risk factors with coronary artery disease (CAD) and generalised vascular illness including hypertension, diabetes, hyperlipidemia, obesity, lack of physical exercise, cigarette smoking, poor diet, excess alcohol intake, and psychological stress [1, 2]. There is consensus to consider all men with ED at risk of cardiovascular disease (CVD) until proven otherwise [3, 4]. An association between erectile dysfunction and ischemic heart disease has been suggested as a consequence of vascular lesions of the penile arteries [5]. Body mass index (BMI) and body fat percentage have been shown to be inversely associated with mortality in patients with CAD [6, 7]. ED is more common among obese men than among men with recommended weight. Studies of patients in clinical settings often include individuals with higher degrees of obesity, with most studies showing a relationship between obesity and lower levels of sexual functioning, especially ED [8]. Fillo et al. [9] found that there is a positive correlation between waist circumference (WC) and ED. Also, ED is common among men with an elevated body mass index (BMI) [10]. Epicardial adipose tissue (EAT) is a visceral fat deposit lying between the myocardium and visceral pericardium and has been thought of as a metabolically active organ that secretes many bioactive molecules [11, 12]. Recently, several studies have demonstrated that epicardial fat is associated with insulin resistance [11], increased cardiometabolic risk [13], inflammatory markers [14, 15], and coronary artery disease [16, 17].

Our aim was to investigate relationships between epicardial adipose tissue (EAT), BMI, WC as cardiometabolic risk factors and erectile dysfunction.

## 2. Materials and Methods

### 2.1. Patients

We selected 30 patients with primary erectile dysfunction and 30 patients without erectile dysfunction. Five-item International Index of Erectile Function (IIEF-5) score was applied to all patients, and IIEF-5 score below 22 was considered as erectile dysfunction [18]. Patients with coronary artery disease, hypertension, atrial fibrillation, left ventricular hypertrophy, endocrine, cerebrovascular, metabolic syndrome, and any medication to cause erectile dysfunction were excluded from study. Echocardiography was performed in all patients to measure epicardial fat thickness. This study was conducted in accordance with the Declaration of Helsinki and was approved by our local ethics committee. Informed consent for the policy was obtained from each patient.

### 2.2. Echocardiography

All patients underwent echocardiography. Epicardial fat thickness was evaluated on the free wall of the right ventricle from the parasternal long-axis view, using the aortic annulus as an anatomic reference. Epicardial fat thickness, identified as an echo-free space between the myocardium and visceral pericardium on two-dimensional echocardiography, was measured perpendicularly, ahead of the right ventricular free wall, at the end of diastole, for three cardiac cycles [19].

### 2.3. Laboratory

Blood samples were drawn by venipuncture to measure routine blood chemistry parameters after fasting for at least eight hours. Fasting blood glucose, serum creatinine, total cholesterol, high-density lipoprotein cholesterol, low-density lipoprotein cholesterol, and triglyceride levels were recorded. Glucose, creatinine, and lipid profile were determined using standard methods.

### 2.4. Statistical Analysis

The statistical analyses were performed using software SPSS 18.0. Parametric values were given as mean ± standard deviation, and nonparametric values were given as a percentage. To compare parametric continuous variables, Student's *t*-test was used; to compare nonparametric continuous variables, the Mann-Whitney *U* test was used. Categorical data were compared by chi-square distribution. Correlation analysis was performed to determine relationship between epicardial fat tissue and another cardiometabolic risk factor. Two-tailed *P* values of less than 0.05 were considered to indicate statistical significance.

## 3. Results

Basal characteristic of patients was shown in Table 1. Age of patients did not differ between two groups. BMI was higher in patients with ED than those without ED (28.19 ± 4.45 kg/m^2^ versus 23.84 ± 2.36 kg/m^2^, *P* = 0.001, resp.). WC was higher in patients with ED than those without ED (106.60 ± 5.90 cm versus 87.86 ± 14.51 cm, *P* = 0.001, resp.). EAT was higher in ED patients compared to non-ED patients (0.49 ± 0.09 cm versus 0.45 ± 0.03 cm, *P* = 0.016,   resp.). TG level was higher in erectile dysfunction patients compared to nonerectile dysfunction patients (115.56 ± 34. 91 mg/dL versus 95.53 ± 29.99 mg/dL, *P* = 0.02) and HDL level was lower in erectile dysfunction patients than control group (38.31 ± 9.1 mg/dL versus 45.96 ± 9.78 mg/dL, *P* = 0.007). But there was no correlation among EAT, TG, and HDL levels. There was a positive correlation between BMI and EAT (*r* : 0.376, *P* = 0.003). Also there was a positive correlation between WC and epicardial fat thickness (*r*: 0.410, *P* = 0.001). There was negative correlation between EFT and IIEF-5 score (*r*: −0.632, *P* = 0.001). Also, BMI and WC were negatively correlated with IIEF-5 score (*r* : −0.753, *P* = 0.001, and *r* : −0.822, *P* = 0.001, resp.)

## 4. Discussion

In the present study, we showed that EAT was higher in erectile dysfunction patients than healthy patients. Also, WC and BMI levels were higher in patients with erectile dysfunction compared to those without erectile dysfunction. Also, epicardial fat tissue was positively correlated with BMI and WC and negatively correlated with IIEF-5 score. Also BMI and WC were positively correlated with IIEF-5 score. These findings suggest that ED is associated with cardiometabolic risk factors including BMI, WC, and EAT and also these factors are associated with severity of ED.

Erectile dysfunction is predominantly a vascular disease and there are well-known modifiable risk factors associated with cardiovascular diseases [20]. There is consensus to consider all men with ED at risk of CV disease until proven otherwise [3, 4]. Many patients present with underlying systemic cardiovascular disease and their first symptom can be erectile dysfunction [21]. One study of 132 men correlated angiographic results with ED symptoms and scores on the 5-item International Index of Erectile Function (IIEF-5); 58% reported experiencing ED before the diagnosis of CHD [22]. Obesity is associated with a high prevalence of erectile dysfunction [23]. Erectile dysfunction (ED) is common among men with an elevated body mass index (BMI) [10]. Also, erectile dysfunction is associated with waist circumstance [10]. Traish et al. suggested that visceral obesity, a component of the metabolic syndrome, adversely affects endothelial function and testosterone levels, leading to hypogonadism and erectile dysfunction [23]. BMI and body fat percentage have been shown to be inversely associated with mortality in patients with CAD [6, 7]. In the present study, our findings were consistent with previous studies. WC and BMI were higher in erectile dysfunction patients compared to nonerectile dysfunction patients. Also, we showed that WC and BMI were associated with severity of ED.

EAT is considered as a surrogate measure of visceral adiposity [19, 24, 25]. EAT has paracrine and endocrine functions. It can secrete numerous bioactive molecules (adipokines) such as adiponectin, resistin, and inflammatory cytokines (interleukin (IL)-1b, IL-6, tumor necrosis factor) [11, 12, 26]. Various studies have emphasized the potential importance of adipose tissue in relation to inflammatory burden in CVD. EFT is clinically related to abdominal visceral adiposity [19], CAD [27], subclinical atherosclerosis [28], and metabolic syndrome [13] and seems to have high capacity of local proinflammatory activity [12]. Mostly cross-sectional or case-control studies have suggested an association between EAT and the risk of subclinical atherosclerosis [29] and CAD [30]. In the present study, we showed that EAT was increased in erectile dysfunction patients compared to those without erectile dysfunction. Also, we showed that EAT is associated with severity of ED. Furthermore, EAT was associated with cardiometabolic risk factor including BMI and WC in erectile dysfunction patients. These findings suggest that ED is associated with cardiometabolic risk factor including BMI, WC, and EAT and this relationship is increasing with severity of ED.

## 5. Limitation 

The main limitation of our study was the small sample size. A small sample size can result in a low statistical power for equivalency testing, leading to false negative results. We could not assess epicardial adipose tissue among mild, moderate, and severe ED patients due to small sample size.

## 6. Conclusion 

EAT, BMI, and WC as cardiometabolic risk factors were higher and associated with severity of ED in erectile dysfunction patients without comorbidities. Patients with erectile dysfunction without comorbidities should be examined carefully for cardiovascular illness.

## Figures and Tables

**Table 1:** Basal characteristic of patients.

	ED(+)	ED(−)	*P* value
Age	46.26 ± 9.32	45.82 ± 8.7	0.713
Glucose mg/dL	94.63 ± 7.90	96.60 ± 9.44	0.385
TG mg/dL	115.56 ± 34.91	95.53 ± 29.99	0.02
LDL mg/dL	113.40 ± 27.48	106.09 ± 34.58	0.368
HDL mg/dL	38.31 ± 9.1	45.96 ± 9.78	0.007
EF%	63.06 ± 3.07	63.26 ± 2.83	0.794
BMI kg/m^2^	28.19 ± 4.45	23.84 ± 2.36	0.001
WC cm	106.60 ± 5.90	87.86 ± 14.51	0.001
EFT cm	049 ± 0.09	0.45 ± 0.03	0.016
IVS cm	0.90 ± 0.08	0.89 ± 0.07	0.546
PW cm	0.85 ± 0.07	0.84 ± 0.07	0.337
IIEF-5	13,83 ± 4,3	24,9 ± 1,12	0,001

TG: triglyceride, HDL: high-density lipoprotein, LDL: low-density lipoprotein, BMI: body mass index, WC: waist circumstance, EFT: epicardial fat thickness, IVS: intraventricular septum, PW: posterior wall, and IIEF-5: 5-item International Index of Erectile Function.
